# Survival and mitochondrial function in septic patients according to mitochondrial DNA haplogroup

**DOI:** 10.1186/cc11150

**Published:** 2012-01-17

**Authors:** Leonardo Lorente, Ruth Iceta, María M Martín, Esther López-Gallardo, Jordi Solé-Violán, José Blanquer, Lorenzo Labarta, César Díaz, Alejandro Jiménez, Julio Montoya, Eduardo Ruiz-Pesini

**Affiliations:** 1 Intensive Care Unit, Hospital Universitario de Canarias, La Laguna 38320, Santa Cruz de Tenerife, Spain; 2 Departamento de Bioquímica y Biología Molecular y Celular, Centro de Investigaciones Biomédicas En Red de Enfermedades Raras (CIBERER), Instituto Aragonés de Ciencias de la Salud (I+CS), Universidad de Zaragoza, Zaragoza 50013, Spain; 3 Intensive Care Unit, Hospital Universitario Nuestra Señora de Candelaria, Santa Cruz de Tenerife 38010, Spain; 4 Intensive Care Unit, Hospital Universitario Dr. Negrín, Las Palmas de Gran Canaria 35010, Spain; 5 Intensive Care Unit, Hospital Clínico Universitario, Valencia 46004, Spain; 6 Intensive Care Unit, Hospital San Jorge, Huesca 22004, Spain; 7 Intensive Care Unit, Hospital Insular, Las Palmas de Gran Canaria 35016, Spain; 8 Research Unit, Hospital Universitario de Canarias, La Laguna 38320, Santa Cruz de Tenerife, Spain; 9 Fundación ARAID, Zaragoza 50013, Spain

## Abstract

**Introduction:**

We recently found that platelet cytochrome c oxidase (COX) activities and quantities in 6-month-survival septic patients are significantly higher than those of patients who died before 6 months. Other studies suggested that the mitochondrial DNA (mtDNA) genotype could play a major role in sepsis survival. Given that COX catalytic subunits are encoded by mtDNA, the objective of the present study was to explore whether mtDNA population genetic variation could affect COX activity and quantity and favors sepsis survival.

**Methods:**

A prospective, multicenter, observational study was carried out in six Spanish ICUs. We included 96 patients with severe sepsis. We determined the mtDNA haplogroup, the COX specific activity/citrate synthase specific activity (COXa/CSa) ratio and the COX quantity/citrate synthase specific activity (COXq/CSa) ratio in circulating platelets at the time of diagnosis, day 4 and day 8. We used survival at 1 and 6 months as endpoints.

**Results:**

Patients with the JT mtDNA haplogroup (*n *= 15) showed higher COXq/CSa ratio at day 4 (*P *= 0.04) and day 8 (*P *= 0.02) than those with other haplogroups (*n *= 81). Logistic regression analysis showed that the JT mtDNA haplogroup (odds ratio = 0.18; 95% confidence interval = 0.04 to 0.94; *P *= 0.04) and COXq/CSa ratio (odds ratio = 0.53; 95% confidence interval = 0.30 to 0.93; *P *= 0.03) were associated with 1-month survival after controlling for age and lactic acid levels.

**Conclusions:**

The novel findings of our study are that 1-month surviving septic patients showed higher COXq/CSa ratio than nonsurviving individuals, that patients from the JT mtDNA haplogroup showed a higher COXq/CSa ratio and that JT patients had a higher 1-month survival than patients from other mtDNA haplogroups.

## Introduction

Sepsis is a common, expensive, and frequently fatal condition [1,2]. The physiopathologic mechanisms of sepsis are not well known, but it has been proposed that organ dysfunction during sepsis is associated with tissue hypoxia due to cellular inability to use oxygen because of mitochondrial dysfunction [3]. Respiratory complex IV or cytochrome c oxidase (COX) is responsible for most cellular oxygen consumption. We have recently found that platelet COX activities and quantities in 6-month-survival patients are significantly higher than those of patients who do not survive 6 months [4]. COX contains 13 polypeptides and three of them are encoded by mitochondrial DNA (mtDNA) [5]. We have also found that transmitochondrial cell lines (cybrids) harboring different mtDNA genetic backgrounds (haplogroups) showed differences in COX activities and quantities. In particular, cybrids from haplogroup H had higher COX activities and quantities than those from haplogroup Uk [6]. Because cybrids are produced by the fusion of cells without mtDNA (rho^0 ^cells) to platelets with mitochondria and mtDNA but lacking a nucleus, different cybrid cell lines contain the same nuclear background and different mitochondrial genotype - therefore, phenotypic differences between them must be due to their particular mtDNA genome. These observations suggest that the mtDNA genotype could play a major role in determining COX quantity and activity and, finally, affecting sepsis survival.

**Figure 1:**
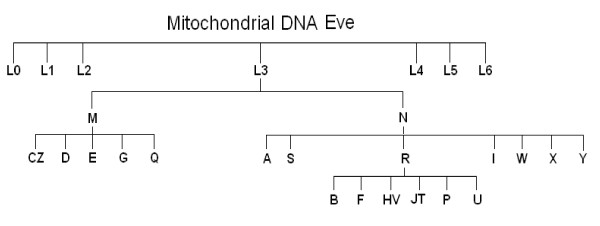
**Human mitochondrial DNA haplogroups**.

Supporting the previous hypothesis, the mtDNA macrolineage R was found in the Chinese Han population to be a strong independent predictor of outcome in severe sepsis, conferring an increased chance of long-term survival [7]. In China, macrolineage R mainly includes haplogroups B and F that represent around 35% of the population. In Europe, this macrolineage includes different haplogroups from those of Asian R. These haplogroups are HV, JT and U (Figure 1), together representing around 90% of the European population [8]. Haplogroup HV contains haplogroup H and is the most prevalent European haplogroup. In England, haplogroup H has also been found to be a strong independent predictor of outcome during severe sepsis, conferring increased chance of survival at 180 days [9].

Chinese and European populations, however, are very different from a mtDNA point of view [10]. Moreover, despite the frequencies of Western Europe mtDNA haplogroups being very similar, the distribution of mtDNA subhaplogroups is probably not so similar [11]. More studies are therefore required to delimitate mtDNA polymorphisms related to sepsis survival. Moreover, a direct intermediate phenotype - linking the relationship between mtDNA haplogroups, mitochondrial function and sepsis survival - has not been analyzed in previous studies. The objective of the present study was thus to explore whether population genetic variation in mtDNA could affect COX activity and quantity, and could modify the risk of sepsis survival.

## Materials and methods

### Design and subjects

An exploratory, prospective, multicenter, observational study was carried out in six Spanish ICUs. Institutional review boards from these hospitals approved the study. Written informed consent was obtained from patients or family members. We determined the mtDNA haplogroup in 96 patients with severe sepsis that have been previously published [4].

The diagnosis of sepsis and severe sepsis was established according to the criteria laid down by the International Sepsis Definitions Conference [12]. Severe sepsis was defined as sepsis complicated by organ dysfunction. Sepsis was defined as a documented or suspected infection and some of the following parameters: general parameters - fever (core temperature > 38.3°C), hypothermia (core temperature < 36.0°C), tachycardia (heart rate > 90 beats/minute), tachypnea (respiratory rate > 30 breaths/minute), altered mental status, significant edema or positive fluid balance (higher than 20 ml/kg over 24 hours), or hyperglycemia (plasma glucose > 110 mg/dl) in the absence of diabetes; inflammatory parameters - leukocytosis (white blood cell count > 12,000/mm^3^), leukopenia (white blood cell count < 4,000/mm^3^), normal white blood cell count with a percentage of immature forms higher than 10%, plasma C reactive protein > 2 standard deviations above the normal value, or plasma procalcitonin > 2 standard deviations above the normal value; hemodynamic parameters - arterial hypotension (systolic blood pressure < 90 mmHg, mean arterial blood pressure < 70 mmHg, or decrease of systolic blood pressure from the baseline > 40 mmHg), mixed venous oxygen saturation > 70%, or cardiac index > 3.5 l/minute/m^2^; organ dysfunction - arterial hypoxemia (PaO_2_/FIO_2 _ratio < 300), acute oliguria (urine output < 0.5 ml/kg/hour for at least 2 hours), increased creatinine ≥ 0.5 mg/dl, thrombocytopenia (platelet count < 100,000/mm^3^), or hyperbilirubinemia (total bilirubin > 4 mg/dl); and tissue perfusion parameters - hyperlactatemia (> 3 mmol/l), decreased capillary refill or mottling. Exclusion criteria were: age < 18 years; pregnancy; lactation; infection by HIV; white blood cell count < 1,000/mm^3^; solid or hematological tumor; or immunosuppressive, steroid or radiation therapy.

The following variables were recorded for each patient: gender, age, diabetes mellitus, chronic obstructive pulmonary disease, ischemic heart disease, ischemic stroke, site of infection, microorganism responsible, bloodstream infection, empiric antimicrobial therapy, mean blood pressure, septic shock, need for and dose of norepinephrine, need for and dose of dobutamine, PaO_2_/FIO_2 _ratio, creatinine, bilirubin, leukocytes, lactic acid, platelets, International Normalized Ratio, activated partial thromboplastin time, Acute Physiology and Chronic Health Evaluation II score [13] and Sepsis-related Organ Failure Assessment score [14]. We used survival at 1 and 6 months as endpoints. Empiric antimicrobial therapy was considered adequate if the microorganism responsible for sepsis was susceptible at least to one antimicrobial agent used.

### Haplogroup classification

Blood samples were collected at the time of diagnosis of severe sepsis. DNA was extracted following standard protocols and the mtDNA haplogroup was determined by real-time PCR. Three SNPs defining haplogroups JT (4216), H (7028) and U (12308) were genotyped in all of the samples. Fourteen other mtDNA SNPs (1811, 3010, 4336, 4580, 4769, 9477, 10873, 13708, 14766, 14793, 14798, 15218, 15257 and 15693) were established using a phylogenetic approach to confirm particular haplogroups (Figure 1) [15]. The real-time PCR was performed with TaqMan reagents (Applied Biosystems, Austin, TX, USA). For each SNP, reagents included two primers around the SNP and two probes: a fluorophore VIC-labeled probe specific for one allele and another fluorophore FAM-labeled probe specific for the other allele. DNA was amplified in a final volume of 25 μl, using 12.5 μl TaqMan Gene Expression Master Mix, 0.9 μM each primer, 0.2 μM each probe and 10 ng total DNA. The amplification was performed using universal conditions.

### Biochemical determination of COX activity and quantity in patients' platelets

Blood samples were collected at the time of diagnosis of severe sepsis, day 4 and day 8. Patients' platelets were obtained according to previously described protocols [16], and protein levels [17] and citrate synthase (CS) specific activity [18] were assayed. We also determined platelets' COX specific activity and quantity by a spectrophotometric assay using Mitoprofile Human Complex IV Activity and Quantity from Mitosciences (Invitrogen, Carlsbad, CA, USA) according to the manufacturer's instructions. This kit immunocaptures complex IV, and the activity is determined by following the oxidation of reduced cytochrome c as an absorbance decrease at 550 nm. Subsequently, in the same well, the quantity of enzyme is measured by adding a complex IV specific antibody conjugated with alkaline phosphatase.

COX activities and quantities are expressed as millioptical density per minute per milligram of protein. The specific activity of CS was also expressed as millioptical density per minute per milligram of protein. To correct for potential differences in the mitochondria number of platelets, we normalized COX specific activities and quantities by CS specific activity (COXact/CSact and COXqua/CSact).

### Statistical analysis

Continuous variables are reported as medians and interquartile ranges. We used a Kruskal-Wallis analysis of variance to compare the four haplogroups before pair comparisons with the Wilcoxon-Mann-Whitney test of continuous variables. Categorical variables are reported as frequencies and percentages, and were compared by chi-square test.

As the COX quantity/CS specific activity and COX specific activity/CS specific activity ratios were not normally distributed, we created two logarithmically transformed variables [19] entitled COXq/CSa and COXa/CSa, respectively:

$$\begin{array}{c} \text{COXa}/\text{CSa}=[\text{log}(\text{COXact}/\text{CSact}]+5;\text{and}\\ \;\;\;\;\text{COXq}/\text{CSa}=[\text{log}(\text{COXqua}/\text{CSact}]+3. \end{array}$$

Logistic regression analyses were used to test the association of JT mtDNA haplogroups and COXq/CSa ratio with survival at 1 and 6 months, controlling for age and lactic acid levels. Odds ratios and their 95% confidence intervals were calculated as measures of the association.

Analysis of survival at 1 and 6 months with the Kaplan-Meier method curve and comparisons by log-rank test were carried out using JT haplogroup versus non-JT mtDNA haplogroup as the independent variable and survival at 1 and 6 months as the dependent variable. *P *< 0.05 was considered statistically significant. When Bonferroni correction was used to correct multiple comparisons in repeated measures between the JT and other haplogroups [20], *P *< 0.008 was considered statistically significant (*p *= 0.05/6 = 0.008).

Statistical analyses were performed using SPSS 17.0 (SPSS Inc., Chicago, IL, USA) and Med Calc (Mariakerke, Belgium).

## Results

We found significant differences between mtDNA haplogroups in COXq/CSa ratios at day 4 (*P *= 0.04) and day 8 (*P *= 0.02) (Table 1). Specifically, patients from the JT mtDNA haplogroup showed a higher COXq/CSa ratio at day 4 than those from the HV (*P *= 0.005) and U (*P *= 0.02) mtDNA haplogroups; and a higher ratio at day 8 than those of the HV (*P *= 0.007) and U (*P *= 0.004) mtDNA haplogroups.

**Table 1:** COXa/CSa and COXq/CSa ratios according to mitochondrial DNA haplogroup

	Haplogroup				
		
	HV	U	JT	No R	*P *value
Time of diagnosis	(*n *= 44)	(*n *= 24)	(*n *= 15)	(*n *= 13)	
COXa/CSa ratio	2.83 (2.34 to 3.17)	2.90 (2.24 to 3.17)	3.25 (2.63 to 3.56)	2.81 (2.39 to 3.09)	0.28
COXq/CSa ratio	1.71 (1.26 to 2.90)	1.89 (1.18 to 2.85)	2.72 (1.57 to 3.38)	2.23 (1.31 to 3.31)	0.41
Day 4	(*n *= 34)	(*n *= 18)	(*n *= 10)	(*n *= 10)	
COXa/CSa ratio	2.73 (2.41 to 3.30)	2.93 (2.45 to 3.16)	3.24 (3.04 to 3.33)	2.85 (2.39 to 3.04)	0.10
COXq/CSa ratio	1.53 (1.26 to 2.99)	2.14 (1.34 to 3.18)	3.10 (2.52 to 3.45)	2.51 (1.23 to 3.34)	0.04
Day 8	(*n *= 32)	(*n *= 14)	(*n *= 10)	(*n *= 9)	
COXa/CSa ratio	2.68 (2.22 to 3.23)	2.72 (2.39 to 3.23)	3.07 (2.83 to 3.15)	3.04 (2.32 to 3.41)	0.41
COXq/CSa ratio	1.59 (1.19 to 3.06)	1.63 (1.21 to 2.79)	3.39 (2.61 to 3.52)	2.91 (1.24 to 3.44)	0.02

Data presented as median (interquartile range). COXa/CSa, cytochrome c oxidase specific activity/citrate synthase specific activity; COXq/CSa, cytochrome c oxidase quantity/citrate synthase specific activity.

Table 2 shows patients' demographic and clinical characteristics according to mtDNA haplogroups. Despite the percentage of 1-month and 6-month survival being higher in JT individuals than in those from other mtDNA haplogroups, we did not find significant differences. Interestingly, there were significant differences in lactic acid levels between these major European haplogroups. The JT haplogroup thus had the higher age and higher lactic acid levels, variables which have been associated with sepsis survival in different studies [1,4,21-24]. A random accumulation of older individuals with higher lactic acid levels from the JT haplogroup in our sample could therefore possibly counteract the beneficial effects of a higher COXq/CSa ratio on survival. Therefore, as we found significant differences between mtDNA haplogroups in COXq/CSa ratios and differences, although not statistically significant, in the survival rate between mtDNA haplogroups in favor of JT mtDNA haplogroup, we included a subanalysis comparing the JT mtDNA haplogroup versus other haplogroups.

**Table 2:** Patients' demographic and clinical characteristics according to mitochondrial DNA haplogroup

	Haplogroup				
		
	HV *(n *= 44)	U *(n *= 24)	JT (*n *= 15)	No R (*n *= 13)	*P *value
Gender, male	28 (63.6)	17 (70.8)	8 (53.3)	7 (53.8)	0.64
Age (years)	57 (46 to 65)	62 (52 to 74)	64 (54 to 81)	50 (39 to 66)	0.06
Diabetes mellitus	21 (47.7)	9 (37.5)	4 (26.7)	2 (15.4)	0.14
COPD	5 (11.4)	3 (12.5)	1 (6.7)	1 (7.7)	0.92
Ischemic heart disease	3 (6.8)	2 (8.3)	1 (6.7)	1 (7.7)	0.99
Ischemic stroke	2 (4.5)	1 (4.2)	0	0	0.73
Site of infection					0.97
Respiratory	31 (70.5)	16 (66.7)	8 (53.3)	7 (53.8)	
Abdominal	8 (18.2)	4 (16.7)	5 (33.3)	4 (30.8)	
Urinary	1 (2.3)	1 (4.2)	1 (6.7)	1 (7.7)	
Skin	2 (4.5)	2 (8.3)	1 (6.7)	1 (7.7)	
Endocarditis	1 (2.3)	1 (4.2)	0	0	
Others	1 (2.3)	0	0	0	
Microorganism responsible					
Unknown	23 (52.3)	13 (54.2)	6 (40.0)	7 (53.8)	0.83
Gram-positive	11 (25.0)	6 (25.0)	5 (33.3)	2 (15.4)	0.75
Gram-negative	9 (20.5)	5 (20.8)	4 (26.7)	4 (30.8)	0.85
Fungi	3 (6.8)	1 (4.2)	0	0	0.62
Anaerobe	1 (0.8)	0	0	0	0.78
Bloodstream infection	5 (11.4)	3 (12.5)	3 (20.0)	2 (15.4)	0.86
Empiric antimicrobial treatment					0.99
Unknown if adequate due to negative cultures	23 (52.3)	12 (50.0)	7 (46.7)	7 (53.8)	
Adequate	17 (38.6)	10 (41.7)	6 (40.0)	5 (38.5)	
Unknown if adequate due to diagnosis by antigenuria	3 (6.8)	2 (8.3)	2 (13.3)	1 (7.7)	
Inadequate	1 (2.3)	0	0	0	
β-lactamic more aminoglycoside	10 (22.7)	5 (20.8)	3 (20.0)	2 (15.4)	0.95
β-lactamic more quinolone	25 (56.8)	14 (58.3)	6 (40.0)	7 (53.8)	0.68
Mean blood pressure (mmHg)	68 (63 to 71)	66 (63 to 71)	69 (63 to 75)	69 (63 to 72)	0.63
Septic shock	38 (86.4)	21 (87.5)	13 (86.7)	11 (84.6)	0.99
Norepinephrine	38 (86.4)	21 (87.5)	13 (86.7)	11 (84.6)	0.99
Norepinephrine dose (μg/kg/minute)	0.5 (0.3 to 0.8)	0.5 (0.3 to 0.7)	0.5 (0.4 to 0.9)	0.5 (0.3 to 1.0)	0.77
Dobutamine	3 (6.8)	2 (8.3)	1 (6.7)	1 (7.7)	0.99
Dobutamine dose (μg/kg/minute)	6.0 (5.0 to 8.0)	6.0 (5.0 to 7.0)	5.0	8.0	0.56
PaO_2_/FIO_2 _ratio	199 (107 to 280)	152 (80 to 250)	138 (84 to 197)	128 (99 to 335)	0.38
Creatinine (mg/dl)	1.20 (0.80 to 2.06)	1.75 (1.00 to 2.17)	2.05 (1.10 to 3.07)	0.75 (0.47 to 1.42)	0.20
Bilirubin (mg/dl)	1.20 (0.64 to 2.30)	1.37 (0.88 to 2.56)	0.92 (0.48 to 1.44)	0.40 (0.17 to 0.66)	0.10
Leukocytes (×10^3^/mm^3^)	12.2 (5.4 to 19.9)	10.2 (5.2 to 20.4)	16.4 (8.5 to 25.9)	13.8 (11.6 to 16.9)	0.81
Lactic acid (mmol/l)	2.35 (1.42 to 4.45)	3.20 (1.30 to 5.87)	3.80 (1.70 to 4.65)	1.30 (0.87 to 2.00)	0.048
Platelets (×10^3^/mm^3^)	166 (86 to 224)	185 (78 to 312)	120 (43 to 193)	207 (103 to 294)	0.30
INR	1.42 (1.11 to 1.65)	1.38 (1.20 to 1.65)	1.24 (1.15 to 1.67)	1.21 (1.07 to 1.52)	0.63
aPTT (seconds)	33 (29 to 45)	35 (31 to 41)	36 (26 to 49)	30 (28 to 34)	0.40
APACHE II score	20 (16 to 25)	20 (14 to 24)	21 (18 to 23)	23 (14 to 29)	0.86
SOFA score	9 (7 to 12)	10 (9 to 14)	9 (8 to 12)	9 (6 to 11)	0.26
Survivors at 180 days	23 (52.3)	13 (54.2)	11 (73.3)	7 (53.8)	0.54
Survivors at 30 days	26 (59.1)	17 (70.8)	13 (86.7)	7 (53.8)	0.18

Data presented as number (percentage) or median (interquartile range). COPD, chronic obstructive pulmonary disease; PaO_2_/FIO_2_, pressure of arterial oxygen/fraction inspired oxygen; aPTT, activated partial thromboplastin time; INR, International Normalized Ratio; APACHE, Acute Physiology and Chronic Health Evaluation; SOFA, Sepsis-related Organ Failure Assessment.

In the analysis of the JT haplotype versus other haplogroups, we found higher COXa/CSa and COXq/CSa ratios at days 1, 4 and 8 in individuals from the JT haplogroup (Table 3). After the Bonferroni correction for multiple analyses, however, only the COXq/CSa ratio at days 4 and 8 was significantly higher in JT individuals. We found a higher, not statistically significant, survival rate in patients from the JT mtDNA haplogroup than in those from other haplogroups at 6 months (73.3% vs. 53.1%; *P *= 0.17) and at 1 month (86.7% vs. 61.7%; *P *= 0.08) (Table 4).

**Table 3:** Comparison of COXa/CSa and COXq/CSa ratios between JT and other mitochondrial DNA haplogroups

	Other haplogroups	JT haplogroup	*P *value
Time of diagnosis	(*n *= 81)	(*n *= 15)	
COXa/CSa ratio	2.84 (2.37 to 3.16)	3.25 (2.63 to 3.56)	0.04
COXq/CSa ratio	1.80 (1.25 to 2.90)	2.72 (1.57 to 3.38)	0.01
Day 4	(*n *= 62)	(*n *= 10)	
COXa/CSa ratio	2.81 (2.43 to 3.19)	3.24 (3.04 to 3.33)	0.01
COXq/CSa ratio	1.67 (1.27 to 3.14)	3.10 (2.52 to 3.45)	0.004*
Day 8	(*n *= 55)	(*n *= 10)	
COXa/CSa ratio	2.72 (2.32 to 3.26)	3.07 (2.83 to 3.15)	0.04
COXq/CSa ratio	1.67 (1.22 to 3.06)	3.39 (2.61 to 3.52)	0.002*

Data presented as median (interquartile range). COXa/CSa, cytochrome c oxidase specific activity/citrate synthase specific activity; COXq/CSa, cytochrome c oxidase quantity/citrate synthase specific activity. *Statistically significant after Bonferroni correction.

**Table 4:** Patients' demographic and clinical characteristics according to mitochondrial DNA haplogroup

	Other haplogroups (*n *= 81)	JT haplogroup (*n *= 15)	*P *value
Gender male	52 (64.2)	8 (53.3)	0.56
Age (years)	59 (48 to 68)	64 (54 to 81)	0.11
Diabetes mellitus	32 (39.5)	4 (26.7)	0.40
COPD	9(11.1)	1 (6.7)	0.99
Ischemic heart disease	6 (7.4)	1 (6.7)	0.99
Ischemic stroke	3 (3.7)	0	0.99
Site of infection			0.96
Respiratory	54 (66.7)	8 (53.3)	
Abdominal	16 (19.8)	5 (33.3)	
Urinary	3 (3.7)	1 (6.7)	
Skin	5 (6.2)	1 (6.7)	
Endocarditis	2 (2.5)	0	
Others	1 (1.2)	0	
Microorganism responsible			
Unknown	43 (53.1)	6 (40.0)	0.41
Gram-positive	19 (23.5)	5 (33.3)	0.52
Gram-negative	18 (22.2)	4 (26.7)	0.74
Fungi	4 (4.9)	0	0.99
Anaerobe	1 (1.2)	0	0.99
Bloodstream infection	10 (12.3)	3 (20.0)	0.42
Empiric antimicrobial treatment			0.81
Unknown if adequate due to negative cultures	42 (51.9)	7 (46.7)	
Adequate	32 (39.5)	6 (40.0)	
Unknown if adequate due to diagnosis by antigenuria	6 (7.4)	2 (13.3)	
Inadequate	1 (1.2)	0	
β-lactamic more aminoglycoside	17 (21.0)	3 (20.0)	0.30
β-lactamic more quinolone	46 (56.8)	6 (40.0)	0.58
Mean blood pressure (mmHg)	68 (63 to 71)	69 (63 to 75)	0.74
Septic shock	70 (86.4)	13 (86.7)	0.99
Norepinephrine	70 (86.4)	13 (86.7)	0.99
Norepinephrine dose (μg/kg/minute)	0.5 (0.3 to 0.8)	0.5 (0.4 to 0.9)	0.75
Dobutamine	6 (7.4)	1 (6.7)	0.99
Dobutamine dose (μg/kg/minute)	6.0 (5.0 to 8.0)	5.0	0.57
PaO_2_/FIO_2 _ratio	160 (102 to 265)	138 (84 to 197)	0.98
Creatinine (mg/dl)	1.20 (0.70 to 2.00)	2.05 (1.10 to 3.07)	0.35
Bilirubin (mg/dl)	1.00 (0.55 to 2.30)	0.92 (0.48 to 1.44)	0.54
Leukocytes (×10^3^/mm^3^)	12.2 (5.4 to 19.9)	16.4 (8.5 to 25.9)	0.82
Lactic acid (mmol/l)	2.20 (1.30 to 4.37)	3.80 (1.70 to 4.65)	0.20
Platelets (×10^3^/mm^3^)	180 (89 to 266)	120 (43 to 193)	0.28
INR	1.32 (1.11 to 1.56)	1.24 (1.15 to 1.67)	0.35
aPTT (seconds)	33 (29 to 42)	36 (26 to 49)	0.30
APACHE II score	20 (16 to 25)	21 (18 to 23)	0.98
SOFA score	9 (7 to 12)	9 (8 to 12)	0.78
Survivors at 180 days	43 (53.1)	11 (73.3)	0.17
Survivors at 30 days	50 (61.7)	13 (86.7)	0.08

Data presented as number (percentage) or median (interquartile range). COPD, chronic obstructive pulmonary disease; PaO_2_/FIO_2_, pressure of arterial oxygen/fraction inspired oxygen; aPTT, activated partial thromboplastin time; INR, International Normalized Ratio; APACHE, Acute Physiology and Chronic Health Evaluation; SOFA, Sepsis-related Organ Failure Assessment.

Logistic regression analyses showed that the JT haplogroup was associated with higher survival at 1 month (odds ratio = 0.18; 95% confidence interval = 0.04 to 0.94; *P *= 0.04) after controlling for age and lactic acid levels (Table 5). Logistic regression analyses also showed that the COXq/CSa ratio was associated with higher survival at 1 month (odds ratio = 0.53; 95% confidence interval = 0.30 to 0.93; *P *= 0.03) and at 6 months (odds ratio = 0.56; 95% confidence interval = 0.34 to 0.93; *P *= 0.03) after controlling for age and lactic acid levels (Table 5).

**Table 5:** Multiple logistic regression analyses to predict mortality

	Odds ratio	95% confidence interval	*P *value
First model: mortality at 1 month as dependent variable			
Mitochondrial DNA haplogroup JT	0.18	0.04 to 0.94	0.04
Lactic acid	1.23	1.05 to 1.44	0.01
Age	1.03	0.99 to 1.06	0. 11
Second model: mortality at 6 months as dependent variable			
Mitochondrial DNA haplogroup JT	0.27	0.07 to 1.03	0.056
Lactic acid	1.29	1.07 to 1.56	0.008
Age	1.03	1.001 to 1.071	0. 04
Third model: mortality at 1 month as dependent variable			
COXq/CSa	0.53	0.30 to 0.93	0.03
Lactic acid	1.17	0.99 to 1.37	0.07
Age	1.03	0.99 to 1.07	0.12
Fourth model: mortality at 6 months as dependent variable			
COXq/CSa	0.56	0.34 to 0.93	0.03
Lactic acid	1.18	0.98 to 1.42	0.08
Age	1.03	1.00 to 1.07	0.052

COXa/CSa, cytochrome c oxidase specific activity/citrate synthase specific activity; COXq/CSa, cytochrome c oxidase quantity/citrate synthase specific activity.

**Figure 2:**
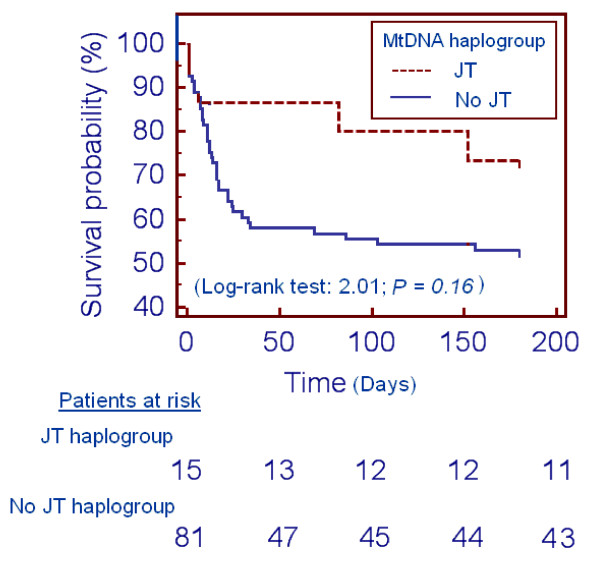
**Association of survival with the JT mitochondrial DNA haplogroup**. Cumulative proportion of surviving patients at 6 months according to the JT mitochondrial DNA (mtDNA) haplogroup versus non-JT mtDNA haplogroup.

We have not been able to demonstrate in Kaplan-Meier analyses that the JT haplogroup was associated with higher survival at 1 month (log-rank test = 2.84; *P *= 0.09) and 6 months (log-rank test = 2.01; *P *= 0.16) (Figure 2).

## Discussion

We had previously found that 6-month survival in sepsis patients was significantly associated with platelet COX quantity [4]. However, 1-month survival is a more frequently used parameter in critically ill patients; and we have found that this parameter is also significantly associated with platelet COX quantity. We had previously observed that COX levels can be determined by mtDNA genetic background [6], and other investigators showed that mtDNA haplogroups modified 6-month survival [9]. Here we show that the mtDNA haplogroup determines the platelet COX quantity in sepsis patients and that those patients from the JT mtDNA haplogroup had higher survival rate than those from other mtDNA haplogroups.

The JT mtDNA haplogroup is defined by polymorphisms in nucleotide positions m.4216T > C/*MT-ND1*, m.11251A > G/*MT-ND4*, m.15452C > A/*MT-CYB *and m.16126T > C/*MT-DLOOP*. The last polymorphism is located in the control region, out of any important sequence for the regulation of mtDNA replication and transcription. M.11251A > G is a synonymous polymorphism. Only m.4216T > C and m.15452C > A provoke amino acid substitutions. The first mutation produces the substitution of tyrosine by histidine in position 304 of ND1 (p.MT-ND1:Y304H), and the second produces a change of leucine by isoleucine in position 236 of cytochrome b (p.MT-CYB:L236I). Supporting our results, the m.4216T allele has been found to be a risk factor for mortality after severe trauma [25]. However, there are no genetic differences between the JT and other haplogroups in respiratory complex IV mtDNA-encoded genes. The question then arises of how can we explain the higher respiratory complex IV levels in platelets of JT individuals with sepsis? Two hypotheses can therefore be advanced.

First, it is now thought that respiratory complexes interact within entities named supercomplexes [26] and it has been shown that the assembly kinetic of respiratory complex IV or supercomplexes is faster in haplogroup J cybrids [27]. Therefore, the first hypothesis is that the previously mentioned polymorphisms in respiratory complex I (p.MT-ND1) and complex III (p.MT-CYB) subunits may exert a protective effect on the stability of respiratory chain complex IV.

Second, the expression of mtDNA genes is proportional to the mtDNA copy number [28] and it has recently been shown that mtDNA depletion occurs in patients with sepsis and correlates with disease severity [29]. When compared with cybrids from haplogroup H, those from haplogroup J showed a significant increase in mtDNA copy number. This increase was apparently due to the presence of a mutation (m.295C > T) in a control region important for mtDNA replication [30]. Therefore, the second hypothesis is that higher mtDNA levels in JT haplogroup individuals could determine higher levels of respiratory complex IV.

In addition to the three genes for respiratory complex IV (*MT-CO1 *to *MT-CO3*), mtDNA also includes seven genes (*MT-ND1 *to *MT-ND-6 *and *MT-ND4L*), one gene (*MT-CYB*) and two genes (*MT-ATP6 *and *MT-ATP8*) for oxidative phosphorylation (OXPHOS) complexes I, III and V, respectively, and the RNAs (22 transfer RNAs and two ribosomal RNAs) required for the expression of those polypeptides. Interestingly, other studies have shown that OXPHOS complex I [31] and OXPHOS complex V [32] are affected in sepsis patients. OXPHOS function has also been found affected in different tissues from different organisms suffering from sepsis [4]. All of these observations suggest a general effect of sepsis on OXPHOS function. Moreover, it has been found that the human blood leukocyte response to acute systemic inflammation includes the modulation of translational machinery and a transient dysregulation of leukocyte bioenergetics. Significant decreases in mRNA abundance were thus observed in the mitochondrial OXPHOS complex I to complex V genes in humans [33], and mtDNA is damaged in murine sepsis models [34-36]. Small OXPHOS differences due to haplogroup-defining mtDNA population polymorphisms can thus determine whether an individual will cross a pathological OXPHOS threshold. Those individuals with a slightly higher OXPHOS function will have a better chance of survival. Particular mtDNA polymorphisms, modifying OXPHOS capacities, therefore appear to contribute to sepsis survival but they are only one of a combination of required factors.

Some limitations of our study must be recognized. First, the causal chain in the theoretical model is a follows: patients with the JT mtDNA haplogroup show increase of COXq normalized by CS; and, finally, COXq normalized by CS is associated with mortality. In our study, only 15 patients have JT mtDNA; thus, the lack of power is a drawback to test a complete model that jointly includes mtDNA, COX specific activity, COX quantity and survival. However, we found an association between mtDNA haplogroups and survival controlling for confounding variables, such as age and lactic acid levels. The concordance between these findings and biochemical data suggest that this genetic association is real. Second, as previously discussed [4], the platelet COX quantity is unlikely to be the factor that determines death in severe sepsis patients, although it probably mirrors the quantity in other tissue and organs - but, because we did not want to use a very invasive protocol, we were not able to correlate this parameter between different organs.

## Conclusion

The novel findings of our study are that 1-month surviving septic patients showed higher COXq/CSa than nonsurviving individuals, that patients from the JT mtDNA haplogroup showed a higher COXq/CSa ratio and that JT patients had a higher 1-month survival than patients from other mtDNA haplogroups.

## Key messages

• One-month-surviving septic patients have a higher platelet COXq/CSa ratio than nonsurvival patients.

• Septic patients with the JT mtDNA haplogroup showed a higher platelet COXq/CSa ratio than those from other mtDNA haplogroups.

• Septic patients with the JT mtDNA haplogroup have higher survival at 1 month than those from other mtDNA haplogroups.

## Abbreviations

COX: cytochrome c oxidase; COXa/CSa: cytochrome c oxidase specific activity/citrate synthase specific activity; COXq/CSa: cytochrome c oxidase quantity/citrate synthase specific activity; CS: citrate synthase; mtDNA: mitochondrial deoxyribonucleic acid; OXPHOS: oxidative phosphorylation; PaO_2_/FIO_2_: pressure of arterial oxygen/fraction inspired oxygen; PCR: polymerase chain reaction; SNP: single nucleotide polymorphism.

## Competing interests

The authors declare that they have no competing interests.

## Authors' contributions

LLo has made substantial contributions to conception and design, acquisition of data, analysis and interpretation of data, drafting the article, revising the article critically for important intellectual content, and final approval of the version to be published. RI, EL-G and JM have made substantial contributions to conception and design, analysis and interpretation of data, revising the article critically for important intellectual content, and final approval of the version to be published. MMM, JS-V, JB, LLa and CD have made substantial contributions to acquisition of data, revising the article critically for important intellectual content, and final approval of the version to be published. AJ has made substantial contributions to analysis and interpretation of data, revising the article critically for important intellectual content, and final approval of the version to be published. ER-P has made substantial contributions to conception and design, analysis and interpretation of data, drafting the article, revising the article critically for important intellectual content, and final approval of the version to be published. All authors read and approved the manuscript for publication.
